# Ultrasound-guided needle tracking with deep learning: A novel approach with photoacoustic ground truth

**DOI:** 10.1016/j.pacs.2023.100575

**Published:** 2023-11-29

**Authors:** Xie Hui, Praveenbalaji Rajendran, Tong Ling, Xianjin Dai, Lei Xing, Manojit Pramanik

**Affiliations:** a School of Chemistry, Chemical Engineering and Biotechnology, Nanyang Technological University, Singapore 637459, Singapore; b Stanford University, Department of Radiation Oncology, Stanford, California 94305, United States; c School of Electrical and Electronic Engineering, Nanyang Technological University, Singapore 637459, Singapore; d Department of Electrical and Computer Engineering, Iowa State University, Ames, IA 50011, United States

**Keywords:** Needle tracking, Ultrasound imaging, Photoacoustic imaging, Deep learning

## Abstract

Accurate needle guidance is crucial for safe and effective clinical diagnosis and treatment procedures. Conventional ultrasound (US)-guided needle insertion often encounters challenges in consistency and precisely visualizing the needle, necessitating the development of reliable methods to track the needle. As a powerful tool in image processing, deep learning has shown promise for enhancing needle visibility in US images, although its dependence on manual annotation or simulated data as ground truth can lead to potential bias or difficulties in generalizing to real US images. Photoacoustic (PA) imaging has demonstrated its capability for high-contrast needle visualization. In this study, we explore the potential of PA imaging as a reliable ground truth for deep learning network training without the need for expert annotation. Our network (UIU-Net), trained on ex vivo tissue image datasets, has shown remarkable precision in localizing needles within US images. The evaluation of needle segmentation performance extends across previously unseen ex vivo data and in vivo human data (collected from an open-source data repository). Specifically, for human data, the Modified Hausdorff Distance (MHD) value stands at approximately 3.73, and the targeting error value is around 2.03, indicating the strong similarity and small needle orientation deviation between the predicted needle and actual needle location. A key advantage of our method is its applicability beyond US images captured from specific imaging systems, extending to images from other US imaging systems.

## 1 Introduction

Needle insertion is a commonly used procedure in clinical diagnosis and therapy, offering a minimally invasive approach to access and treat various medical conditions [1], [2], [3], [4], [5]. Needle insertion is always used to obtain tissue samples for accurate diagnosis, guide the delivery of medications or fluids to specific targets, and perform therapeutic interventions. In diagnosis, procedures such as fine-needle aspiration biopsy (FNSB) and core needle biopsy rely on needle penetration to collect samples for pathological examination and determine the presence or nature of diseases [1], [2]. Needle insertion is also employed in therapeutic treatments like injection, central venous catheter placements, and radiofrequency ablations, allowing for the precise delivery of medications, fluids, or radio waves. These procedures involve inserting a metallic needle into the body towards the target region, guided in real-time by various imaging modalities including ultrasound (US) imaging, X-ray computer tomography (CT), and magnetic resonance imaging (MRI). Among the available options, US imaging is the predominant choice for visualizing the needle and surrounding tissue to ensure safe procedures [3], [6]. While the ultrasound-guided needle insertion process boasts several advantages such as real-time imaging capabilities, cost-effectiveness, non-invasiveness, and the absence of ionizing radiation, it does have certain limitations. US imaging can struggle with visualizing needle penetration into tissue due to depth-dependent attenuation and the inherent angular dependency. These factors restrict the transducer's ability to fully record needle echoes, thereby reducing contrast [7]. If the needle is not adequately visualized, even experienced operators may encounter complications such as nerve damage or organ injury [8]. This emphasizes the crucial role of proper needle guidance (through visualization or any other form of feedback) in mitigating potential risks associated with needle insertion.

Efforts to enhance the US-assisted needle insertion process have led to several innovative improvements. One notable advancement involves surface modifications of the needles, achieved through methods like coating [9], [10] and laser-etching [11]. These techniques can significantly enhance the visibility of the needle in US images, as evident from the promising results reported in the literature. Nonetheless, these modifications often require specialized manufacturing processes, which can be resource intensive. In addition to physical modifications, signal and image-processing-based techniques have also been developed to improve needle visibility. These include methods like the Hough Transform [12], [13], Gabor filtering [14], Random Sample Consensus (RANSAC) [15], [16], and graph cut [17]. While these techniques have demonstrated potential, applying these techniques in real-time ultrasound scanning scenarios with inhomogeneous backgrounds can be challenging. Moreover, achieving a balance between computation time and accuracy using these methods can be complex.

Over the past two decades, deep learning has emerged as a powerful tool for tackling a wide range of signal and image processing tasks, making it increasingly prevalent in the field of medical imaging. Convolutional Neural Network (CNN) based architectures are most extensively employed to effectively address complex imaging challenges with robustness and efficiency. This has led to their application in enhancing needle visualization in ultrasound images by leveraging the power of data [18], [19], [20], [21], [22]. For instance, Zhao *et al.*, have utilized the attention U-net to accurately localize needles within US images, achieving a precision rate of 96.25% [23]. Despite the impressive results achieved, deep learning-based methods suffer from the requirements of large datasets with precise needle annotations for effective training, which is challenging to obtain in practice. Simulated data, often used as a substitute, may not translate well to real clinical situations despite providing known needle locations [24]. Images from ex vivo or in vivo experiments can also serve as training datasets, while the true needle locations remain elusive. Manual annotations by domain experts offer an alternative but can be labor-intensive and potentially introduce bias [25], [26]. Therefore, accurate and automatic segmentation of the needle is necessary.

Photoacoustic (PA) imaging, which combines optical illumination and acoustic detection, has gained increasing attention for its potential in preclinical and clinical applications [27], [28], [29], [30], [31], [32], [33]. PA imaging has the capability to image deep tissue with high spatio-temporal resolution and contrast, complementing US imaging [34], [35], [36]. Recent studies have utilized dual US/PA imaging to offer mutually beneficial information. US imaging provides valuable insights into tissue structure and PA imaging identifies critical tissue and surgical devices like needles [37], [38], [39]. Unlike US imaging, which often provides poor image contrast and requires expertise to track the needle, PA imaging provides high-contrast needle visualization, due to the strong light absorption by the metallic needle. The exceptional capability of PA imaging in needle localization has spurred studies on needle detection and enhancement of needle visibility in PA imaging through deep learning [39]. However, many existing PA systems are not yet suitable for clinical use due to factors such as the use of nonclinical US transducers and immobile system design [40]. Many dual-modal US+PA imaging systems are still in research mode without FDA clearance for real clinical use, although recently, a pre-market approval (PMA) from The Food and Drug Administration (FDA) was granted to an opto-acoustic ultrasound system for diagnostic breast cancer imaging [41]. In addition, the use of high-energy class IV lasers inside the clinic adds another layer of safety concern and makes it even more challenging for clinical translation. Hence, commercially available clinical ultrasound imaging systems remain the preferred choice by clinicians for their day-to-day routine practices. Therefore, despite the challenges PA has [42], with its superior needle visualization capabilities, PA imaging can play an important role in precise needle localization under US-guided needle insertion procedures.

In this study, the high-contrast needle visibility in PA images was used as ground truth (without the need for any domain expert to annotate the needle in the ultrasound images, as done in traditional methods) to train a deep learning neural network, UIU-Net, to augment the needle signal in US images. Ex vivo experiments were conducted by inserting a needle into chicken tissue at various depths and angles, while US and PA images were simultaneously stored using a clinical dual modal US+PA imaging system. The preprocessed PA images served as valuable ground truth for training the neural network, bypassing the need for expert annotation. The trained network was then applied to US images from an independent ex vivo experiment and an open-source in vivo human US image [43]. This method's adaptability to US images (obtained with different imaging systems) makes it suitable for clinical use with a wide variety of existing clinical ultrasound systems. Although the method was demonstrated using specific US systems' datasets, it possesses enough generalizability to be applied to US images from other imaging systems. This is the first time PA imaging is used as a training tool to augment needle visualization in US imaging (through deep learning). Therefore, without being in the clinic (yet) PA imaging can still be used to enhance and augment the clinical ultrasound imaging capabilities.

## 2 Materials and methods

### 2.1 Network implementation

**Fig. 1:**
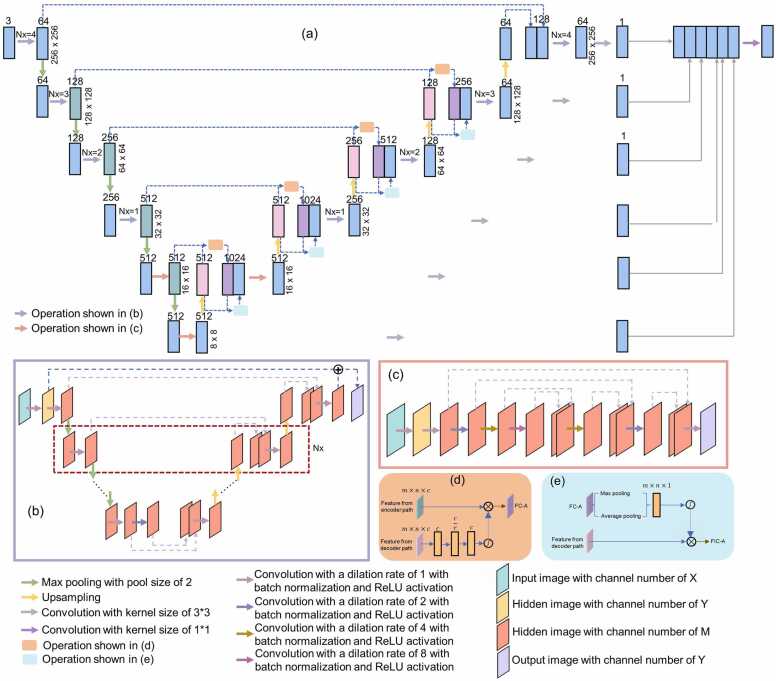
Network architecture for improving needle visibility in US imaging. (a) The backbone architecture of UIU-Net, (b) the architecture of small U-Net which is incorporated into the backbone, represented by lilac arrows in (a), (c) the architecture of small U-Net which is incorporated into the backbone in the last two scales, represented by light red arrows in (a), (d) detailed structure of IC-A module yielding cross channel feature F_C-A_, represented by skin-color boxes in (a), (e) detailed structure of IC-A module yielding interactive-cross spatial attention feature F_IC-A_, represented by light blue boxes in (a).

The implemented neural network, UIU-Net, was an extension of the U-Net architecture [44], designed specifically for image segmentation tasks, with its architecture shown in Fig. 1(a). Unlike traditional U-Nets, UIU-Net integrated a smaller U-Net into a larger U-Net backbone, enabling multi-level and multi-scale feature extraction. This architecture improved object segmentation accuracy for small-sized objects and limited datasets. Additionally, UIU-Net incorporated two modules: the resolution-maintenance deep supervision (RM-DS) module and the interactive-cross attention (IC-A) module, which enhanced global and local context representation, respectively. In the RM-DS module, ReSidual U-blocks (RSUs) were integrated into a deep network to maintain resolution. This approach improved the ability of the RSU-based U-Net to extract the object's global features. The IC-A module, embedded in the RM-DS network, captured long-range dependencies between pixel-based objects by interactively cross-coding low-level details and high-level semantic features. It replaced the skip layer in U-Net. The final output of UIU-Net was the fusion of the multi-layer output from the RM-DS network. The encoder path of the backbone consisted of multiple scales, each containing a small U-Net with varying depth (represented as Nx). The small U-Net at each scale is shown in Fig. 1(b). A 2 × 2 max pooling layer followed each scale. In the last two scales, the small U-Net was replaced by a series of convolutions with different dilation rates [Fig. 1(c)]. The decoder path also followed a similar structure, with each scale (except the bottom two scales) containing a small U-Net followed by a transposed convolution layer with an up-sampling factor of 2. After each up-sampling, the interactive-cross attention module encoded the high-level and low-level features from the encoder path, capturing more context information from the decoder layer [Fig. 1(d-e)].

In the assessment of processing multiple US frames for dynamic needle tracking, the effectiveness of the UIU-Net was examined in relation to other widely used U-Net variants, namely conventional U-Net [45], Attention U-Net [46], and R2U-Net [47]. The conventional U-Net, originating as the foundational U-Net architecture, employs a symmetric encoder-decoder design that precisely extracts features while constraining the number of trainable parameters. Despite its success across diverse segmentation tasks, it may struggle with detecting fine details and distinguishing between closely located structures. Furthermore, susceptibility to noise and artifacts can precipitate inaccuracies in segmentation outcomes. Addressing these limitations, the Attention U-Net emerges as a formidable contender by incorporating attention gates, empowering the network to selectively focus on the target structures within the input images. However, this attention mechanism may not always capture all relevant contextual information, leaving the model susceptible to imprecision when exposed to noise or intricate structures. In contrast, the R2U-Net takes a distinct approach, introducing recurrent connections into the U-Net framework. This innovation significantly enhances the model's capacity to leverage long-range contextual information from the input image, further bolstering its segmentation capabilities. In this study, the conventional U-Net, Attention U-Net, and R2U-Net were enlisted as reference models to assess and compare the performance of needle tracking in relation to the UIU-Net.

All the examined networks, including UIU-Net and the three reference U-nets, were trained under the same setting, including input image size, learning rate, optimization algorithm, number of iterations, and batch size. The model training process employed input pairs with a resolution of 256 × 256 pixels. The networks were implemented in Python using Pytorch v1.2.0. For optimizing the networks, the Adam optimization algorithm decouple the weight decay (AdamW) was used. The learning rate was set to 0.001, and networks were trained on a Nvidia Tesla V100–32 GB GPU using the nodes of the Gekko cluster, High-Performance Computing Centre, Nanyang Technological University, Singapore. The UIU-Net employed a multi-binary cross-entropy (BCE) loss function, which is analogous to the one employed in the original implementation [44], and the loss function employed for the U-Net, Attention U-Net, and R2U-Net was the typical binary cross-entropy (BCE) loss. All networks were trained for 20000 iterations of training with a batch size of 2 and the model weights with the lowest validation loss were saved.

### 2.2 Imaging system description

**Fig. 2:**
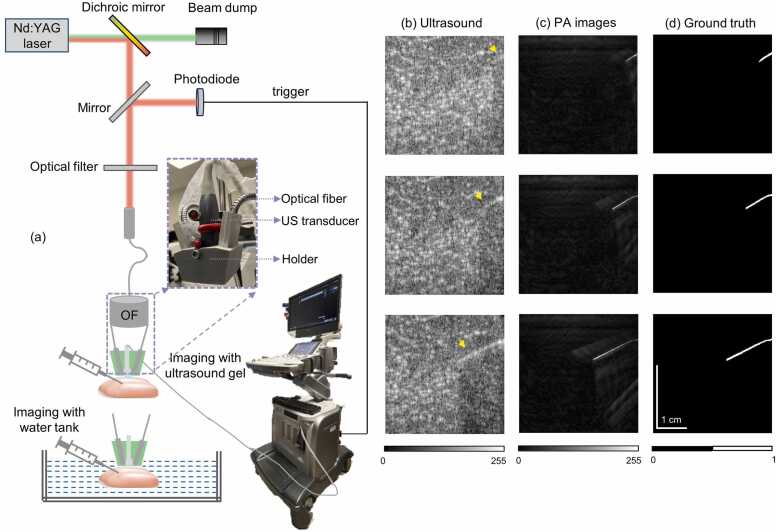
Imaging system descriptions and representative US/PA images. (a) Imaging systems used to capture both US and PA images. Inset: photograph of the handheld probe combining optical fiber, US transducer, and the holder, OF optical fiber. (b) Representative US images during needle insertion, where yellow arrows mark the needle position. (c) Representative PA images during needle insertion. (d) Preprocessed PA images which served as ground truth for network training. (b-d) Have same scale bar as shown in one of the figures.

To obtain the training data sets, both US images and the corresponding PA images (which act as the ground truth, without any manual expert annotation needed), a dual-modal US+PA imaging system was used. As shown in Fig. 2(a), the imaging system utilized a frequency-doubled nanosecond pulsed Nd:YAG pump laser (Continuum, Surelite Ex) to generate laser pulses with a pulse repetition frequency of 10 Hz and a pulse width of 5 ns. The laser pulses passed through a dichroic mirror (HBSY12, Thorlabs), resulting in a 1064-nm beam reflection and a 532-nm beam transmission. The transmitted 532-nm light was directed toward the beam dump (LB2/M, Thorlabs). The reflected 1064-nm light beam was split by a glass slide into two beams, with one beam used as a trigger for the clinical research US system (ECUBE 12 R, Alpinion, South Korea) by passing through a photodiode, and the other directed to an optical filter designed to remove light with unwanted wavelengths. After passing through the filter, the light beam was coupled and transmitted to the bifurcated optical fiber bundle (Ceramoptec CmbH, Germany). The fiber bundle was composed of 1600 optical fibers, each with a core diameter of 185 µm and a numerical aperture of 0.22. The fibers were bifurcated at the center to evenly spread over two rectangular regions with the size of 4 cm × 0.1 cm. The resulting two rectangular fiber bundles, and the L3–12 linear array ultrasound transducer of the clinical US system, were assembled into a handheld probe holder (made using a Dremel 3D20 3D printer). Through Monte Carlo simulations, various probe holder parameters were optimized for the imaging process, considering light delivery and needle visualization by PA imaging [48]. The illumination angle of the light for this holder was 15 degrees. Considering that the total illumination area from both ends was around 3 cm^2^, the fiber bundle’s 25% coupling and transmission efficiency, and the energy per pulse was ∼100 mJ, the fluence on the sample surface was calculated to be approximately 8.3 mJ/cm^2^. This value was well below the safety limit established by the American National Standards Institute (ANSI) [49].

The US and PA imaging data were acquired using the clinical research US system. This system could perform US and PA imaging either separately or simultaneously in dual mode, and save three types of data, including raw radio frequency (RF) data for each channel, beamformed data, and IQ data. For this study, we exclusively employed the dual mode (research mode) and acquired only the beamformed data. The system utilized a 128-element linear array transducer with a width of each element of 0.03 cm, a center frequency of 8 MHz and a 95% fraction bandwidth. In dual mode, 128 transmission channels and 64 receive channels were configured. The system employed 64 parallel data acquisition hardware to collect data per laser pulse. As a result, to store data from all 128 channels and generate a complete PA image, two laser pulses were necessary. This resulted in a frame rate of 5 frames per second for the imaging system, despite the laser operating at 10 Hz. Various parameters were specified using Python code, such as imaging depth and number of frames to save. The imaging depth and sound speed was set to 2 cm and 1540 m/s, respectively, and the maximum number of frames to save at a time was set to 500. This setting allowed for recording up to 100 s of the scanning at one go.

### 2.3 Acquisition of ex vivo image

Both US and PA data were acquired during needle insertions into ex vivo chicken tissue. The experiments were conducted under two conditions, one with a water tank and another with an ultrasound gel applied to the transducer for better ultrasound coupling, as depicted in Fig. 2(a). We continuously recorded the insertion of a 1.2 mm × 38 mm 18 G needle (BD PrecisionGlide Needle, Franklin Lakes, NJ, USA) into the chicken tissue at various angles and depth of insertion [with the needle being inserted parallel to the longer side of the ultrasound transducer (In-plane)]. As the imaging system (in research mode) allows users to save beamformed US and PA data, we did not do any specific reconstruction on our own. We only read the beamformed data in MATLAB and use it for further network training purposes. To obtain the US images from beamformed data, first quadratic demodulation was done, which involved multiplying each captured modulated signal with a reference signal of fixed frequency, synchronized with the carrier frequency of the modulated signal. Following this, a low-pass filter was applied, and envelop signals were obtained. Given the wide dynamic range of amplitudes inherent in US signals, log compression was employed to narrow the dynamic range.

Three representative US images are shown in Fig. 2(b), where images at different time points during the needle insertion are shown in different rows. The corresponding PA images, obtained from the beamformed data via the Hilbert transform, are displayed in Fig. 2(c). Although the high background US signal from surrounding tissue made it difficult to observe the needle or track its trajectory in the US images [marked with yellow arrows in Fig. 2(b)], the needle is clearly visible with very high contrast in PA images [Fig. 2(c)]. This is possible due to the strong optical absorption (and PA images provide optical absorption contrast) of the needle compared to the background tissue, producing strong PA signals from the needle. However, some laser absorption artifacts from surrounding chromophores are also present in the PA images, although not significantly affecting the needle visualization. To optimize the neural network training by feeding higher-contrast PA images, several pre-processing steps were performed, including thresholding, binarization, connected component labeling, and selecting the large area of the connected regions. The preprocessing process was automated for all acquired PA images, resulting in the generation of binary PA maps, as illustrated in Fig. 2(d). The US images and binary PA maps, corresponding to a field-of-view of 3.81 (lateral) x 1.89 (axial) cm, were used for the network training, with the PA images serving as ground truth. Detailed videos (s1, s2, and s3) demonstrating needle insertion in US imaging, PA imaging, and preprocessed PA imaging are available in the supplementary materials. Approximately 2600 US frames and corresponding PA frames, captured from 11 experiments, were divided in the ratio of 80:10:10 into training, validation, and test datasets, respectively, for the training of the neural network. In clinical scenarios, a wide range of needles with different lengths and gauges (diameters) are available. To ensure the proposed network’s feasibility in clinical situations, it must have the capability to generalize across different needle diameters. Therefore, we also performed similar experiments and collected data from 23 G needles (BD PrecisionGlide Needle, Franklin Lakes, NJ, USA) to test the performance of the network. Here, we employed identical experimental setups and acquisition settings, while using a new chicken tissue sample.

### 2.4 Evaluation of in vivo image

In vivo needle insertions into the human body were sourced from an open-source data repository, which offered US images and clips illustrating needle insertion into various areas such as the breast and axilla, kidney, salivary gland, and parotid gland [43]. The US images featuring in-plane placements with a single needle were considered, and the needle locations have been indicated by the author. Based on the annotated needle location, lines which connected the needle tip and the needle base served as needle segmentation maps (labels) for subsequent comparison. These in vivo images, acquired from Fujifilm Ultrasound, were applied to the trained network for automatic needle localization, and the results were compared with the manual segmentation map. By generalizing the model for in vivo US images, the network's feasibility for clinical application can be assessed.

### 2.5 Evaluation metrics

As noted in prior literature, traditional deep learning evaluation concentrated on pixel-wise segmentation accuracy, which may disregard some biologically relevant instances [50]. In this study, we employed a modified Hausdorff distance (MHD) that was derived from the Hausdorff distance and quantified the similarity between two sets of edge maps related to the objects under consideration. Due to its enhanced discriminatory capability and robustness to outliers, it was superior to other distance measures for the purpose of object matching. Considering two sets of points $A=\left\{a_{1},a_{2},\ldots ,a_{{\mathrm{Num}}_{A}}\right\}$ and $B=\left\{b_{1},b_{2},\ldots ,b_{{\mathrm{Num}}_{B}}\right\}$ of the desired objects from two different images, the distance between a point from A and B was defined as ${d\left(a,B\right)=\min}_{b\in B}\left\|a-b\right\|$. Thus, the distance between two points sets A and B con be defined as

$$\mathrm{MHD}=\max\left(d\left(A,B\right),d\left(B,A\right)\right)=\max\left(\frac{1}{{\mathrm{Num}}_{A}}\sum_{a\in A}d\left(a,B\right),\frac{1}{{\mathrm{Num}}_{B}}\sum_{b\in B}d\left(b,A\right)\right)$$

MHD was defined in pixel units, considering the consistent pixel size of the images utilized in this research.

In addition, two validation metrics called needle localization success rate (NLSR) and targeting error (TE) were employed to assess the ability of the proposed algorithm to localize needles [51], [52]. NLSR quantifies the percentage of images where the localization result accurately identifies the needle. A successful localization is needed to fulfill the following requirements: 1) the line determined by the localization step is required to pass through the ground truth, and 2) the measured segmentation outcome should be in close agreement with the ground truth segmentation. The set of needle pixels from ground truth was denoted as SG, while the set of needle pixels predicted by the network was denoted as SH. To ensure the above criteria are met, the intersection of SG and SH should exceed a specified threshold.

TE can be considered a clinically pertinent measure related to targeting specific structures, and its value is strongly influenced by orientation errors. TE was defined as

$$\mathrm{TE}=\left|d_{\mathrm{true}}-d_{\mathrm{segmentation}}\right|$$

where, $d_{\mathrm{true}}$ represents the minimum distance between the actual needle line and the central pixel of the image, while $d_{\mathrm{segmentation}}$ refers to the minimum distance between the estimated needle line and the image’s central pixel. To avoid distortion of TE statistics due to significant inaccuracies in localization, TE was calculated exclusively using successful outcomes.

In addition to needle orientation error, the needle length ratio was also computed by dividing the predicted needle length by the actual needle length. The ratio was defined as

$$\mathrm{Needle}\;\mathrm{length}\;\mathrm{ratio}=\frac{\mathrm{Predicted}\;\mathrm{needle}\;\mathrm{length}}{\mathrm{Actual}\;\mathrm{needle}\;\mathrm{length}}$$

The needle length was determined using the 2D Euclidean distance between the needle tip and the base of the needle shape in the image, providing a more complete assessment of the network's performance in detecting needles. When the needle length ratio approaches closer to 1, it signifies a higher degree of completeness in the needle prediction. It should be noted that only successful localization will be further calculated MHD, TE, and needle length ratio.

## 3 Results

### 3.1 Deep neural network training, validation, and testing

**Fig. 3:**
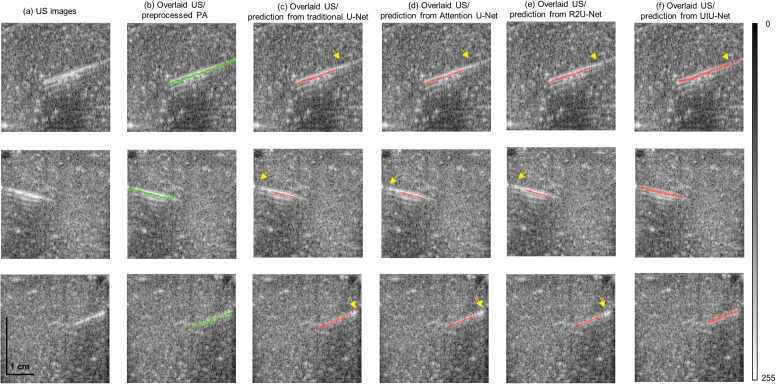
Representative images from the test dataset with needle insertions into chicken tissue. (a) conventional US images, (b) overlaid US image with preprocessed PA (served as ground truth), (c) overlaid US image with the prediction from traditional U-Net, (d) overlaid US image with the prediction from Attention U-Net, (e) overlaid US image with the prediction from R2U-Net, (f) overlaid US image with the prediction from UIU-Net. All figures have same scale bar, as shown in (a).

To showcase the effectiveness of needle enhancement in US images, the trained network was applied to US images not utilized in training or validation. Fig. 3(a) presents three representative test US images, while the corresponding overlaid US with PA image (which acts as the ground truths) are shown in Fig. 3(b). The ground truth here served only as reference images for subsequent quantitative assessment. Different network predictions are shown in Fig. 3(c-f). This side-by-side comparison revealed that the needle can be detected by the trained U-Net or its extension network, although segmentation performance varied. There was no significant difference in the needles predicted by the traditional U-Net, Attention U-Net, and R2U-Net. Using the three networks, the needles can be identified in the US images, but not the entire needle was detected. These networks tended to accurately localize the needle tip rather than the needle root, which has been marked by yellow arrows [in Fig. 3(c-e)]. However, compared with these U-Net variants, UIU-Net [Fig. 3(f)] outperformed by precisely localizing the entire needle despite some discontinuity present in the prediction result as marked by a yellow arrow.

Table 1 shows the quantitative results of the performance of all these different networks on the test data set. Using the average value of each metric across test images as an indicator of overall performance, all employed networks demonstrate 100% confidence, as shown by NLSR value, in localizing the needle. The mean MHD values for traditional U-Net, Attention U-Net, and R2U-Net all fell within the range of 2.86–3.27. Slightly contrasting this, the UIU-Net model achieved a lower mean MHD value of 1.90. Notably, the standard deviations associated with the MHD values of the three reference networks exceed 3.99, significantly higher than that of UIU-Net (1.10). This not only underscores the stable performance of UIU-Net but also indicates that the other three networks sometimes generated needle maps that diverged significantly from the actual needle map. This divergence is primarily attributed to the fact that predicted needle maps from the reference networks often omit parts of the needle, as visibly observed in Fig. 3(c-e). Furthermore, the mean targeting errors between the actual needle and the predicted needle from the four examined networks did not display significant disparities. The largest variation among the mean targeting errors for these networks did not surpass a margin of 1. This implies that despite the incompleteness of predictions made by the reference networks, all four networks predicted needles with similar orientations. The incompleteness in the predictions made by the reference networks was further underscored by the needle length ratio. The mean needle length ratio obtained from UIU-Net was 92.99%, significantly higher than the ratios obtained from the other examined networks, all of which fell below 87%. Also, the standard deviation of the needle length ratio produced by UIU-Net was 9.27%, distinctly outperforming the other networks, each of which exceeded 13%. This outcome further substantiates the superior needle localization performance of UIU-Net, as it consistently detects nearly the entire length of the needle.

**Table 1:** Quantitative assessment of the needle segmentation in prediction results. The metrics (MHD, targeting error, and needle length ratio), expressed as mean ± standard deviations, were taken from test sets.

	NLSR	MHD	Targeting error	Needle length ratio
Prediction from traditional U-Net	100%	3.17 ± 4.59	2.92 ± 3.01	86.37% ± 14.37%
Prediction from Attention-Net	100%	3.27 ± 4.72	3.39 ± 3.55	84.36% ± 15.22%
Prediction from R2U-Net	100%	2.86 ± 3.99	3.70 ± 4.24	85.12% ± 13.89%
Prediction from UIU-Net	100%	1.90 ± 1.10	3.57 ± 6.59	92.99% ± 9.27%

### 3.2 Enhanced needle visibility on unseen ex vivo tissue US images

**Fig. 4:**
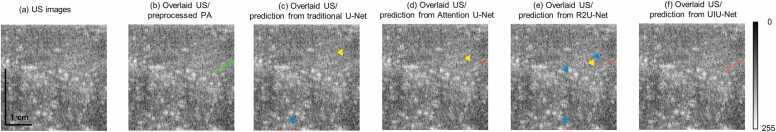
Representative images from a separate experiment (unseen data) with 18 G needle insertions into chicken tissue. (a) conventional US images, (b) overlaid US image with preprocessed PA (served as ground truth for comparison), (c) overlaid US image with the prediction from traditional U-Net, (d) overlaid US image with the prediction from Attention U-Net, (e) overlaid US image with the prediction from R2U-Net, (f) overlaid US image with the prediction from UIU-Net. All figures have same scale bar, as shown in (a).

To verify the network’s robustness on previously unseen data, an additional experiment involving the insertion of an 18 G needle was conducted. The US images captured from the needle insertion procedure were fed into the network to assess its performance without relying on ground truth annotations. A representative of the captured US image is depicted in Fig. 4(a). Correspondingly, the overlaid US/PA image is shown in Fig. 4(b). Overlaid US images with predicted needle generated by the employed U-Net architectures are shown in Fig. 4(c-f).

The traditional U-Net, Attention U-Net, and R2U-Net models appeared to exhibit comparatively reduced effectiveness in accurately detecting needles, in contrast to the UIU-Net model [Fig. 4(f)]. Instances of needle misidentification were evident in the traditional U-Net’s predictions, particularly at image boundaries, marked by blue arrows in Fig. 4(c). R2U-Net displayed similar instances of needle misidentification, both at image boundaries and within bright regions within the image, as denoted by the blue arrow in Fig. 4(e). In addition to misidentification, the structural integrity of the needles remained inadequately preserved in the predictions from the three reference networks, most notably in the U-Net's outcomes where needles were completely missed. This was marked by yellow arrows in Fig. 4(c-e), indicating the needles that went undetected by the network. Although both the Attention U-Net and R2U-Net demonstrated slightly better performance compared to U-Net, they still exhibited susceptibility to ignoring the needles, capturing only a limited portion of their structure. Conversely, the outcomes generated by the UIU-Net showcased its adeptness in precisely identifying needle locations while offering comprehensive segmentation of the needle structures [Fig. 4(f)]. Supplementary Material S4 depicted the process of needle insertion in US images, while S5 showed the improved visibility of needles in US images achieved through the application of UIU-Net.

In the previous evaluation of the test dataset, UIU-Net's needle localization capability did not exhibit a substantial improvement compared to the reference networks. However, as shown in Table 2, the quantitative results presented for this new, previously unseen dataset suggest that the reference networks' ability to generalize was not as strong as that of UIU-Net, as they failed to effectively detect the needles in this unfamiliar dataset. Among the four U-Nets, convention U-Net displayed the worst evaluation metric values, which was consistent with the observations in Fig. 4. This was followed by Attention U-Net and R2U-Net, both of which showed worse quantitative measurements compared with UIU-Net. Specifically, U-Net's NLSR value was notably low at approximately 93.55%, indicating its limited ability to robustly track needles, with around 6% of needles remaining undetected. A higher NLSR can be achieved by using Attention U-Net and R2U-Net (94.76% and 94.96%, respectively), but they still displayed a tendency to miss needles in some US images. In contrast, UIU-Net achieved NLSR value of about 99.80%, a visible improvement over the reference models, with only 0.2% of needles being missed.

**Table 2:** Quantitative assessment of the needle segmentation in prediction results. The metrics (MHD, targeting error, and needle length ratio), expressed as mean ± standard deviations, were taken from a separate experiment with 18 G needle insertions into chicken tissue.

	NLSR	MHD	Targeting error	Needle length ratio
Prediction from traditional U-Net	93.55%	8.29 ± 8.73	20.59 ± 16.53	66.09% ± 25.03%
Prediction from Attention-Net	94.76%	6.33 ± 8.83	17.35 ± 17.08	70.18% ± 23.08%
Prediction from R2U-Net	94.96%	4.13 ± 6.58	13.44 ± 13.86	79.19% ± 19.72%
Prediction from UIU-Net	99.80%	0.79 ± 1.77	3.21 ± 5.29	95.37% ± 12.13%

For the assessment of network performance in terms of proximity to the actual needle, needle orientation accuracy, and needle length accuracy, we calculated the MHD, targeting error, and needle length ratio using only prediction results that successfully detected the needle. The mean MHD value for UIU-Net (∼0.79) was greatly lower than that of the other U-Nets (over 4), representing a more than fivefold decrease. In addition, UIU-Net’s mean targeting error was less than 3.5, a substantial improvement compared to the reference models where values exceeded 14. The pronounced needle orientation errors evident in the reference models not only highlight a marked disparity between the predicted and actual needle orientations but also signify that these networks can only detect a limited portion of the needles, highlighting a notable incompleteness in needle segmentation. The inability of the reference U-Nets to preserve needle structural integrity is further reflected by the needle length ratio. UIU-Net's mean needle length ratio demonstrated a marked advantage over the reference models, with an approximate 95.37% ratio, in contrast to the considerably lower ratios, all < 80%, associated with the reference models. This discrepancy emphasizes UIU-Net's superior capability in preserving needle integrity. Furthermore, the consistent and stable performance of UIU-Net in needle localization was evident through the relatively small standard deviation observed across all metrics evaluated for the results generated by UIU-Net.

### 3.3 Enhanced visibility of needles with varying diameters

**Fig. 5:**
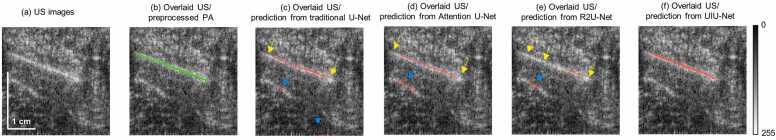
Representative images from a separate experiment (unseen data) with 23 G needle insertions into chicken tissue. (a) conventional US images, (b) overlaid US image with preprocessed PA (served as ground truth for comparison), (c) overlaid US image with the prediction from traditional U-Net, (d) overlaid US image with the prediction from Attention U-Net, (e) overlaid US image with the prediction from R2U-Net, (f) overlaid US image with the prediction from UIU-Net. All figures have same scale bar, as shown in (a).

In clinical applications, it is common to employ needles with different diameters. To assess the applicability of the networks for such clinical scenarios, an additional experiment involving the insertion of a 23 G needle was carried out. Crucially, the data generated from this experiment was excluded from the network's training dataset. Thus, this data can also be considered “unseen” by the network. In Fig. 5(a), a representative US image captured during the experiment is displayed, while Fig. 5(b) presents the corresponding overlaid image combining the US and PA images. Further insights can be gleaned from Fig. 5(c-h), which exhibit overlaid US images with predicted needle generated by various network models.

As illustrated in Fig. 5(c-h), traditional U-Net, Attention U-Net, and R2U-Net showed suboptimal performance in the task of needle detection. While these three U-Nets could localize the needles, they struggled to segment the complete needle, often missing either the needle tip or root, as indicated by the yellow arrows. R2U-Net, in particular, overlooked a substantial portion of the needles. Additionally, they frequently misclassified surrounding tissue as a needle, marked by blue arrows [in Fig. 5(c-e)]. In the case of the U-Net, it even exhibited inaccuracies in delineating the image boundaries as part of the needle. In contrast, UIU-Net effectively segmented a more comprehensive needle while accurately differentiating between needle-like tissue and the actual needle, demonstrating superior performance in needle detection tasks.

Table 3 presents the quantitative results of different networks. Although the NLSR for all four networks were the same, with a value of 100%, the MHD value, targeting error, and needle length ratio varied among them. R2U-Net exhibited a mean MHD value above 22, whereas both traditional U-Net and Attention U-Net had mean MHD values around 7. These values were notably higher than that of UIU-Net, which showed a mean MHD value of around 2.2. The comparatively larger mean MHD value and standard deviation observed in the reference networks highlight a more significant deviation between the actual needle map and the predicted needle map when compared to UIU-Net. The mean targeting error for all assessed networks ranged between 1.79 and 3.30, with U-Net showing the lowest values and R2U-Net the highest. Although UIU-Net did not achieve the lowest mean targeting error, the difference from other networks was slight. In addition, the needle length ratio of UIU-Net demonstrated a value of around 95%, much closer to 1 than that of the other networks (all lower than 86%). Particularly striking was the remarkably low needle length ratio of 44.65% generated by R2U-Net, aligning with the observed needle detection shortcomings in Fig. 5(e).

**Table 3:** Quantitative assessment of the needle segmentation in prediction results. The metrics (MHD, targeting error, and needle length ratio), expressed as mean ± standard deviations, were taken from a separate experiment with 23 G needle insertions into chicken tissue.

	NLSR	MHD	Targeting error	Needle length ratio
Prediction from traditional U-Net	100%	7.12 ± 3.30	1.79 ± 1.10	78.17% ± 10.08%
Prediction from Attention-Net	100%	6.44 ± 4.62	2.40 ± 0.54	85.43% ± 12.12%
Prediction from R2U-Net	100%	22.20 ± 5.39	3.30 ± 2.32	44.65% ± 16.89%
Prediction from UIU-Net	100%	2.20 ± 0.65	2.74 ± 0.62	95.25% ± 14.73%

### 3.4 Application of the UIU-Net on the in vivo US images

**Fig. 6:**
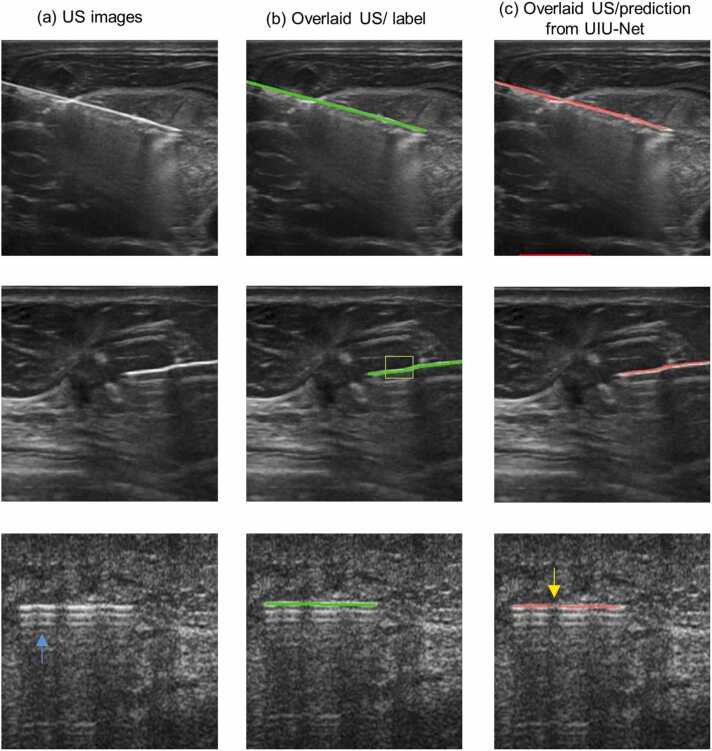
Representative images with needle insertions into the human body. (a) conventional US images, (b) overlaid US image with ground truth (manual label shown in green), (c) overlaid US image with prediction from UIU-Net.

In the preceding sections, UIU-Net not only demonstrated remarkable efficacy in identifying needles within ex vivo datasets but also showcased its robustness in extending its needle localization capability to datasets it had not encountered before. A subsequent evaluation was conducted using UIU-Net on an in vivo human imaging dataset to further validate its reliability and potential utility in clinical settings. The trained model was applied to images obtained from an open-source repository depicting needle insertions into the human body. In Fig. 6(a), conventional US images are presented, while Fig. 6(b) display overlaid US images and ground truth (manual segmentation to evaluate the performance of the UIU-Net). Overlaid US images and predictions from UIU-Net are shown in Fig. 6(c).

As illustrated in Fig. 6, UIU-Net displayed outstanding effectiveness in improving needle visibility, reliably detecting nearly the entire length of the needle, although a slight missing of the needle tip was observed. This phenomenon can be attributed to the relatively sharper nature of the needle tip in the in vivo US images, a feature not well-represented in the ex vivo dataset. Furthermore, in vivo images were not utilized for training the network, thus there might not be enough data in the training dataset that represented this feature, affecting the ability of the UIU-Net to detect the sharp needle tip for the in vivo cases. Despite the minor tip absence, the remaining portion of the needle was well segmented, even when the needle displayed non-linear features within the image. While needles in ex vivo images typically displayed a linear appearance, in vivo human US images sometimes displayed needles with potential non-linearities, denoted by the green box [Fig. 6(b)]. Remarkably, UIU-Net adeptly accommodated this complexity and accurately delineated the presence of the needle. Additionally, during needle insertion into the human body, the interaction between US waves and the needle could lead to the generation of multiple echoes, manifesting as parallel lines in the US image, as pointed out by the blue arrow in the second row of images. However, UIU-Net maintained its resilience and effectively predicted the actual needle while excluding other needle-like features present in the image. Table 4.

**Table 4:** Quantitative assessment of the needle segmentation in prediction results. The metrics (MHD, targeting error, and needle length ratio), expressed as mean ± standard deviations, were taken from in vivo human datasets.

	NLSR	MHD	Targeting error	Needle length ratio
Prediction from UIU-Net	100%	3.73 ± 4.76	2.03 ± 2.21	83.55% ± 13.48%

As shown in Fig. 6(c), the prediction results from UIU-Net robustly outlined the needle with different insertion depths and angles, despite some discontinuity as indicated by yellow arrows. Importantly, these discontinuities did not significantly compromise the precision of needle identification, a fact supported by quantitative results. The mean MHD value and targeting error were recorded at approximately 3.73 and 2.03, respectively. These values denoted a robust alignment between the predicted and actual needle maps, facilitated by the minimal proximity and orientation discrepancy between the two maps. Furthermore, the needle length ratio approximated 83.55%, illustrating UIU-Net’s capacity to predict 83.55% of the needle length even in previously unencountered human datasets from completely different clinical US imaging system data.

An additional assessment of the inference time for the UIU-Net was conducted, considering both ex vivo and in vivo images. The result showed that the average inference time for an ex vivo image with the RTX A5500 GPU (10,240 tensor cores) was 14.4 ms (71 frames/second), while the average inference time for an in vivo image with the RTX A5500 GPU was 37 ms (27 frames/second). The inference times can be further reduced by employing techniques such as data parallelism and model parallelism as well as higher-end GPUs.

## 4 Discussion

Accurate needle guidance is crucial for safe and effective clinical diagnosis and treatment procedures. US-guided needle insertion often encounters challenges in consistency and precise visualization of the needle. Moreover, the operator’s dependency on US imaging makes it vulnerable to a shortage of skilled and experienced ultra-sonographers, especially in resource-crunch rural areas. Therefore, a self-learned Artificial Intelligence (AI)-assisted US scanner with the ability to visualize the needle-tracking precisely will substantially improve the accuracy and safety of needle-based procedures. Moreover, these developments will help to have auto-navigating (in conjunction with robotic arms) US-guided needle tracking in the future, potentially eliminating the need for a skilled and experienced ultra-sonographer. Nonetheless, the performance of AI networks is highly dependent on the training data and the ground truth. While the concept of a self-learning AI-assisted ultrasound (US) scanner offers the promise of reducing the necessity for manual annotation, it’s noteworthy that manual annotation often remains a requisite component when training the AI network [25], [26], [53]. Although certain studies have endeavored to mitigate this challenge by employing simulation datasets, the ability to generalize simulation data to real-world clinical scenarios continues to pose a significant challenge [24].

The proposed technology is the first application of PA imaging to serve as deep learning ground truth for US imaging. While PA imaging has emerged as a technique capable of providing high-contrast visualization of needles, the translation of PA to clinics is challenging for various reasons. However, with this approach, PA imaging still can play a role, even without being inside the clinic. Training the DL algorithm with PA images eliminates manual annotations by experts. Therefore, reliability and consistency in the ground truth improve. Using a reliable ground truth will help the algorithm to be trained better. The developed network, UIU-Net, along with three other reference U-Nets, was initially trained on ex vivo image datasets and subsequently evaluated on multiple datasets, including a test dataset, two previously unseen ex vivo datasets, and an in vivo human dataset. While UIU-Net and the reference networks exhibited comparable performance on the test dataset, it became evident that the reference networks struggled to maintain precise needle localization when faced with previously unseen datasets. In contrast, UIU-Net consistently demonstrated outstanding performance in accurately localizing needles within US images, showcasing its robust generalization capabilities. Specifically, the MHD value and targeting error value in human data stand at about 3.73 and 2.03. These values serve as crucial metrics - MHD measures the proximity between the predicted and actual needle maps, while the targeting error metric assesses the accuracy of needle orientation. Furthermore, the needle's structural integrity finds assurance through the utilization of the UIU-Net architecture. This assertion is supported by the needle length ratio, which closely approaches unity, indicating the model's proficiency in preserving the needle's complete form.

The proposed algorithm not only performed well on needle identification but also showed good generalization capability on unseen US images. Even within in vivo human images captured by different systems, UIU-Net still showed impressive needle segmentation results. It suggests that the developed algorithm holds promise to be easily applied to needle-tracking procedures performed by any other ultrasound machine, making the proposed idea truly system-independent. Using this novel approach to enhance US imaging with PA imaging can impact several clinical procedures where accurate needle location needs to be visualized. The unique combination of AI tools and, in the future, AI-driven robotic scanning will significantly impact the standard-of-care in hospitals [54]. AI-driven US-guided needle tracking can reduce operator dependence, increase accuracy in needle target position, achieve faster target localization (reduce workflow duration), and improve patient comfort.

Our current work is only the start of this journey. There are some limitations and further improvements will be needed. The frame rate of our US system is intrinsically tied to the laser repetition rate. To elevate the frame rate, there are two potential solutions: employing a higher repetition rate laser or reducing the imaging area by employing just 64 data-acquisition channels. In a previous demonstration, this system achieved up to 7000 frames per second for PA imaging [55]. A substantial increase in frame rate holds promising prospects, as it could facilitate the creation of a larger image database, thereby contributing to more effective AI training. In addition, the success of the PA ground truth approach heavily relies on the availability of high-quality PA imaging data. A better approach can be further developed to ensure the needle integrity and total removal of artifacts in PA images. To use a US imaging platform for PA imaging, several probe holders were proposed to integrate the light delivery with the US probe. Among the various holder options considered, we chose the holder with 15 degrees of light delivery to achieve high-quality PA images. Limited by the practical challenges of the fiber bundle, holders with light delivery angles exceeding 15 degrees have not been fabricated yet [48]. In future investigations, it is essential to broaden the scope by developing holders with a wider range of illumination angles to thoroughly assess the optimal angle that yields the highest PA image quality. Besides, a more effective signal processing-based method can be proposed for eliminating artifacts from the PA images. Furthermore, considering the wide range of needle sizes used in clinical applications for different procedures, it is better to incorporate needles of all available diameters in the network evaluation. This comprehensive approach ensures a more robust demonstration of the superior performance of the proposed method.

## 5 Conclusion

In this work, we introduced a deep learning approach for precise needle tracking in US procedures, utilizing photoacoustic images as the ground truth. Our innovative photoacoustic-driven deep learning model was developed using ex vivo data, eliminating the need for expert annotation and reducing the workload and potential for subjective bias. The model performance was assessed on previously unexamined ex vivo tissue data and in vivo human data, exhibiting significantly enhanced needle visualization. The method demonstrated excellent generalizability for tracking needles of varying gauges. Consequently, this deep learning-based approach has the potential to improve minimally invasive procedures that involve percutaneous needle insertions by accurately identifying the needle location.

## Funding

This research did not receive any specific grant from funding agencies in the public, commercial, or not-for-profit sectors.

## Declaration of Competing Interest

The authors declare that they have no known competing financial interests or personal relationships that could have appeared to influence the work reported in this paper.

## Data Availability

data and code shared and link is provided in the manuscript.

## References

[1] Heslin M.J., Lewis J.J., Woodruff J.M., Brennan M.F. (1997). Core needle biopsy for diagnosis of extremity soft tissue sarcoma. Ann. Surg. Oncol..

[2] Amedee R.G., Dhurandhar N.R. (2001). Fine‐needle aspiration biopsy. Laryngoscope.

[3] Chapman G.A., Johnson D., Bodenham A.R. (2006). Visualisation of needle position using ultrasonography. Anaesthesia.

[4] Fischer G.S., Deguet A., Csoma C., Taylor R.H., Fayad L., Carrino J.A., Zinreich S.J., Fichtinger G. (2007). MRI image overlay: application to arthrography needle insertion. Comput. Aided Surg..

[5] Orebaugh S.L., McFadden K., Skorupan H., Bigeleisen P.E. (2010). Subepineurial injection in ultrasound-guided interscalene needle tip placement. Reg. Anesth. Pain. Med..

[6] Arif M., Moelker A., van Walsum T. (2018). Needle tip visibility in 3D ultrasound images. Cardiovasc. Interv. Radiol..

[7] Hovgesen C.H., Wilhjelm J.E., Vilmann P., Kalaitzakis E. (2022). Echogenic surface enhancements for improving needle visualization in ultrasound: a PRISMA systematic review. J. Ultrasound Med..

[8] Chin K.J., Perlas A., Chan V.W., Brull R. (2008). Needle visualization in ultrasound-guided regional anesthesia: challenges and solutions. Reg. Anesth. Pain. Med..

[9] Bergin D., Pappas J.N., Hwang J.J., Sheafor D.H., Paulson E.K. (2002). Echogenic polymer coating: does it improve needle visualization in sonographically guided biopsy?. Am. J. Roentgenol..

[10] Beigi P., Salcudean S.E., Ng G.C., Rohling R. (2021). Enhancement of needle visualization and localization in ultrasound. Int. J. Comput. Assist. Radiol. Surg..

[11] Park J.W., Cheon M.W., Lee M.H. (2016). Phantom study of a new laser-etched needle for improving visibility during ultrasonography-guided lumbar medial branch access with novices. Ann. Rehabil. Med..

[12] Ding M., Fenster A. (2003). A real‐time biopsy needle segmentation technique using Hough Transform. Med. Phys..

[13] Zhou, H., W. Qiu, M. Ding, and S. Zhang. Automatic needle segmentation in 3D ultrasound images using 3D improved Hough transform. in Medical Imaging 2008: Visualization, Image-Guided Procedures, and Modeling. 2008. Proc SPIE.

[14] Kaya M., Bebek O. (2014). 2014 IEEE International Conference on Robotics and Automation (ICRA).

[15] Uherčík M., Kybic J., Liebgott H., Cachard C. (2010). Model fitting using RANSAC for surgical tool localization in 3-D ultrasound images. IEEE Trans. Biomed. Eng..

[16] Waine M., Rossa C., Sloboda R., Usmani N., Tavakoli M. (2015). 2015 IEEE International Conference on Robotics and Automation (ICRA).

[17] Ayvaci A., Yan P., Xu S., Soatto S., Kruecker J. (2011). Biopsy needle detection in transrectal ultrasound. Comput. Med. Imaging Graph..

[18] Pourtaherian A., Ghazvinian Zanjani F., Zinger S., Mihajlovic N., Ng G.C., Korsten H.H., de With P.H. (2018). Robust and semantic needle detection in 3D ultrasound using orthogonal-plane convolutional neural networks. Int. J. Comput. Assist. Radiol. Surg..

[19] Arif M., Moelker A., van Walsum T. (2019). Automatic needle detection and real-time bi-planar needle visualization during 3D ultrasound scanning of the liver. Med. Image Anal..

[20] Zhang Y., Tian Z., Lei Y., Wang T., Patel P., Jani A.B., Curran W.J., Liu T., Yang X. (2020). Automatic multi-needle localization in ultrasound images using large margin mask RCNN for ultrasound-guided prostate brachytherapy. Phys. Med. Biol..

[21] Mwikirize C., Nosher J.L., Hacihaliloglu I. (2018). Convolution neural networks for real-time needle detection and localization in 2D ultrasound. Int. J. Comput. Assist. Radiol. Surg..

[22] Gillies D.J., Rodgers J.R., Gyacskov I., Roy P., Kakani N., Cool D.W., Fenster A. (2020). Deep learning segmentation of general interventional tools in two‐dimensional ultrasound images.. Med. Phys..

[23] Zhao, Y., Y. Lu, X. Lu, J. Jin, L. Tao, and X. Chen. Biopsy Needle Segmentation using Deep Networks on inhomogeneous Ultrasound Images. in 2022 44th Annual International Conference of the IEEE Engineering in Medicine & Biology Society (EMBC). 2022. IEEE. 10.1109/EMBC48229.2022.9871059 36086307

[24] Maneas E., Hauptmann A., Alles E.J., Xia W., Vercauteren T., Ourselin S., David A.L., Arridge S., Desjardins A.E. (2021). Deep learning for instrumented ultrasonic tracking: from synthetic training data to in vivo application. IEEE Trans. Ultrason., Ferroelectr., Freq. Control.

[25] Xu Y., Mo T., Feng Q., Zhong P., Lai M., Eric I., Chang C. (2014). 2014 IEEE international conference on acoustics, speech and signal processing (ICASSP).

[26] Andersén C., Rydén T., Thunberg P., Lagerlöf J.H. (2020). Deep learning‐based digitization of prostate brachytherapy needles in ultrasound images.. Med. Phys..

[27] Zhou Y., Yao J., Wang L.V. (2016). Tutorial on photoacoustic tomography. J. Biomed. Opt..

[28] Wang L.V., Yao J. (2016). A practical guide to photoacoustic tomography in the life sciences. Nat. Methods.

[29] Das D., Pramanik M. (2019). Combined ultrasound and photoacoustic imaging of blood clot during microbubbles-assisted sonothrombolysis.. J. Biomed. Opt..

[30] Kalva S.K., Upputuri P.K., Pramanik M. (2019). High-speed, low-cost, pulsed-laser-diode-based second-generation desktop photoacoustic tomography system. Opt. Lett..

[31] Das D., Sharma A., Rajendran P., Pramanik M. (2021). Another decade of photoacoustic imaging. Phys. Med. Biol..

[32] Hui X., Mohammad M.O.A., Pramanik M. (2022). Looking deep inside tissue with photoacoustic molecular probes: a review. J. Biomed. Opt..

[33] Yao J., Wang L.V. (2021). Perspective on fast-evolving photoacoustic tomography. J. Biomed. Opt..

[34] Wu Z., Li L., Yang Y., Hu P., Li Y., Yang S.Y., Wang L.V., Gao W. (2019). A microrobotic system guided by photoacoustic computed tomography for targeted navigation in intestines in vivo. Sci. Robot..

[35] Nyayapathi N., Xia J. (2019). Photoacoustic imaging of breast cancer: a mini review of system design and image features. J. Biomed. Opt..

[36] Das D., Sivasubramanian K., Rajendran P., Pramanik M. (2021). Label-free high framerate imaging of circulating blood clots using a dual modal ultrasound and photoacoustic system. J. Biophotonics.

[37] Sivasubramanian K., Periyasamy V., Pramanik M. (2018). Non-invasive sentinel lymph node mapping and needle guidance using clinical handheld photoacoustic imaging system in small animal. J. Biophotonics.

[38] Xia W., Kuniyil Ajith Singh M., Maneas E., Sato N., Shigeta Y., Agano T., Ourselin S., West S.J., Desjardins A.E. (2018). Handheld Real-Time LED-Based Photoacoustic and Ultrasound Imaging System for Accurate Visualization of Clinical Metal Needles and Superficial Vasculature to Guide Minimally Invasive Procedures. Sensors.

[39] Shi M., Zhao T., West S.J., Desjardins A.E., Vercauteren T., Xia W. (2022). Improving needle visibility in LED-based photoacoustic imaging using deep learning with semi-synthetic datasets. Photoacoustics.

[40] Kim J., Park S., Jung Y., Chang S., Park J., Zhang Y., Lovell J.F., Kim C. (2016). Programmable real-time clinical photoacoustic and ultrasound imaging system. Sci. Rep..

[41] Inc., S.M.I. FDA Approves Seno Medical’s Ground-Breaking Breast Cancer Diagnostic Technology. [cited 2023 15 November]; Available from: https://senomedical.com/newsroom/press-releases/newsroom-press-releases-2021-fda-approves-seno-medicals-ground-breaking-breast-cancer-diagnostic-technology.

[42] Assi H., Cao R., Catelino M., Cox B., Gilbert F.J., Grohl J., Gurusamy K., Hacker L., Ivory A.M., Joseph J., Knieling F., Leahy M.J., Lilaj L., Manohar S., Meglinski I., Moran C., Murray A., Oraevsky A.A., Pagel M.D., Pramanik M., Raymond J., Singh M.K.A., Vogt W.C., Wang L., Yang S., IPASC Mo, Bohndiek S.E. (2023). A review of strategic roadmapping exercise to advance clinical translation of photoacoustic imaging: From current barriers to future adoption. Photoacoustics.

[43] SonoSkills, F.H.E.a. Ultrasound cases.info. [cited 2023 24 August]; Available from: 〈https://www.ultrasoundcases.info/〉.

[44] Wu X., Hong D., Chanussot J. (2022). UIU-Net: U-Net in U-Net for infrared small object detection. IEEE Trans. Image Process..

[45] Ronneberger, O., P. Fischer, and T. Brox. U-net: Convolutional networks for biomedical image segmentation. in Medical Image Computing and Computer-Assisted Intervention–MICCAI 2015: 18th International Conference, Munich, Germany, October 5–9, 2015, Proceedings, Part III 18. 2015. Springer.

[46] Oktay O., Schlemper J., Folgoc L.L., Lee M., Heinrich M., Misawa K., Mori K., McDonagh S., Hammerla N.Y., Kainz B. (2018). Attention u-net: Learning where to look for the pancreas. arXiv.

[47] Alom, M.Z., M. Hasan, C. Yakopcic, T.M. Taha, and V.K. Asari, Recurrent residual convolutional neural network based on u-net (r2u-net) for medical image segmentation. arXiv, 2018. 1802.06955.

[48] Sivasubramanian K., Periyasamy V., Wen K.K., Pramanik M. (2017). Optimizing light delivery through fiber bundle in photoacoustic imaging with clinical ultrasound system: Monte Carlo simulation and experimental validation. J. Biomed. Opt..

[49] ANSI, American National Standard for Safe Use of Lasers ANSI Z136.1–2022 (American National Standards Institute, Inc., New York, NY, 2022). 2022.

[50] Caicedo J.C., Roth J., Goodman A., Becker T., Karhohs K.W., Broisin M., Molnar C., McQuin C., Singh S., Theis F.J. (2019). Evaluation of deep learning strategies for nucleus segmentation in fluorescence images. Cytom. Part A.

[51] Hatt C.R., Ng G., Parthasarathy V. (2015). Enhanced needle localization in ultrasound using beam steering and learning-based segmentation. Comput. Med. Imaging Graph..

[52] Gao J., Liu P., Liu G.-D., Zhang L. (2021). Robust needle localization and enhancement algorithm for ultrasound by deep learning and beam steering methods. J. Comput. Sci. Technol..

[53] Lin X., Shi H., Fan X., Wang J., Fu Z., Chen Y., Chen S., Chen X., Chen M. (2023). Handheld interventional ultrasound/photoacoustic puncture needle navigation based on deep learning segmentation. Biomed. Opt. Express.

[54] Gao S., Wang Y., Ma X., Zhou H., Jiang Y., Yang K., Lu L., Wang S., Nephew B.C., Fichera L., Fischer G.S., Zhang H.K. (2023). Intraoperative laparoscopic photoacoustic image guidance system in the da Vinci surgical system. Biomed. Opt. Express.

[55] Sivasubramanian K., Pramanik M. (2016). High frame rate photoacoustic imaging at 7000 frames per second using clinical ultrasound system. Biomed. Opt. Express.

